# Epidemiological trends of sepsis in the twenty-first century (2000–2013): an analysis of incidence, mortality, and associated costs in Spain

**DOI:** 10.1186/s12963-018-0160-x

**Published:** 2018-02-12

**Authors:** Alejandro Álvaro-Meca, María A. Jiménez-Sousa, Dariela Micheloud, Ainhoa Sánchez-Lopez, María Heredia-Rodríguez, Eduardo Tamayo, Salvador Resino, on behalf of the Group of Biomedical Research in Critical Care Medicine (BioCritic)

**Affiliations:** 1 Departamento de Medicina Preventiva y Salud Pública, Facultad de Ciencias de la Salud, Universidad Rey Juan Carlos, Alcorcón, Madrid Spain; 2 Unidad de Infección Viral e Inmunidad, Centro Nacional de Microbiología, Instituto de Salud Carlos III, Majadahonda, Madrid Spain; 3 Servicio de Urgencias, Hospital General Universitario “Gregorio Marañón”, Madrid, Spain; 4 Departamento de Anestesiología y Reanimación, Hospital Clínico Universitario, Valladolid, Spain

**Keywords:** Sepsis, Mortality, Case fatality rate, Epidemiology, Cost, Hospital resources, Length of hospital stay

## Abstract

**Background:**

Sepsis has represented a substantial health care and economic burden worldwide during the previous several decades. Our aim was to analyze the epidemiological trends of hospital admissions, deaths, hospital resource expenditures, and associated costs related to sepsis during the twenty-first century in Spain.

**Methods:**

We performed a retrospective study of all sepsis-related hospitalizations in Spanish public hospitals from 2000 to 2013. Data were obtained from records in the Minimum Basic Data Set. The outcome variables were sepsis, death, length of hospital stay (LOHS), and sepsis-associated costs. The study period was divided into three calendar periods (2000–2004, 2005–2009, and 2010–2013).

**Results:**

Overall, 2,646,445 patients with sepsis were included, 485,685 of whom had died (18.4%). The incidence of sepsis (events per 1000 population) increased from 3.30 (2000–2004) to 4.28 (2005–2009) to 4.45 (2010–2013) (*p* < 0.001). The mortality rates from sepsis (deaths per 10,000 population) increased from 6.34 (2000–2004) to 7.88 (2005–2009) to 7.89 (2010–2013) (p < 0.001). The case fatality rate (CFR) or proportion of patients with sepsis who died decreased from 19.1% (2000–2004) to 18.4% (2005–2009) to 17.9% (2010–2013) (p < 0.001). The LOHS (days) decreased from 15.9 (2000–2004) to 15.7 (2005–2009) to 14.5 (2010–2013) (p < 0.001). Total and per patient hospital costs increased from 2000 to 2011, and then decreased by the impact of the economic crisis.

**Conclusions:**

Sepsis has caused an increasing burden in terms of hospital admission, deaths, and costs in the Spanish public health system during the twenty-first century, but the incidence and mortality seemed to stabilize in 2010–2013. Moreover, there was a significant decrease in LOHS in 2010–2013 and a decline in hospital costs after 2011.

**Electronic supplementary material:**

The online version of this article (10.1186/s12963-018-0160-x) contains supplementary material, which is available to authorized users.

## Background

Sepsis is a life-threatening dysfunction of the organs caused by a dysregulated host response to infection. It is the primary cause of death from infection, especially if it is not recognized and treated promptly [1]. Sepsis has represented a substantial health care and economic burden worldwide during the last decades for several reasons [2, 3]. Firstly, sepsis affects millions of people around the world each year and is the main cause of death among critically ill patients [4–6]. The incidence of sepsis varies across the world. The incidence of severe sepsis has been estimated at around 300 cases per 100,000 population in the United States, and half of these patients are treated in the intensive care unit (ICU) [4]. The incidence of severe sepsis in Sweden in 2005 was 430 per 100,000 population [7]. Additionally, in clinical cohort studies in ICUs, the incidence of sepsis is 11.8% in Australia and New Zealand [8], 14.6% in France [9], 27.1% in the United Kingdom [10], and 30% in the SOAP study [5], which included 198 European ICUs. In most developed countries, sepsis has progressively increased during the last decades [6]. Secondly, patients with sepsis tend to require high resource expenditure in the hospital and the costs of sepsis are quite substantial [2, 3]. The estimated cost per episode in the US is between $20,000 and $50,000; however, the level of cost is related to age, severity of illness, intensive care unit admission, and number of unscheduled surgical procedures, among others [4, 11–14]. The total sepsis-related cost in the US has been estimated at $16 to $25 billion annually [4, 11]. However, there is growing evidence on the impact of the economic crisis on hospital care utilization after 2008 [15–17], but there is little information about the impact of the economic crisis on critical care utilization.

The rise in sepsis rates has promoted national and global efforts to improve awareness, early recognition, diagnosis, and management [18]. The Surviving Sepsis Campaign was developed in 2004 to promote guidelines and performance-improvement practices with the objective of reducing deaths from sepsis worldwide [19]. This has meant that despite the increase in sepsis incidence, the case fatality rate (CFR) has significantly decreased in most developed countries [2, 20–24]. However, the CFR remains higher than for heart failure and other important pathologies such as breast cancer, colon cancer, and AIDS [25]. Despite these data, there is limited information on the epidemiology of sepsis in Europe in the twenty-first century [7, 24, 26].

The aim of this study was to analyze the epidemiological trends of hospital admissions, deaths, hospital resource expenditures, and associated costs related to sepsis from 2000 to 2013 in Spain.

## Methods

### Study design and data source

We carried out a nationwide population-based retrospective study of all hospitalizations involving sepsis in Spanish public hospitals between January 1, 2000 and December 31, 2013.

Data were obtained from records in the Minimum Basic Data Set (MBDS) of the National Surveillance System for Hospital Data in Spain, provided by the Ministry of Health Social Services and Equality (MSSSI). The MBDS is a clinical and administrative database containing clinical information recorded at the time of hospital discharge, which has an estimated coverage of 92% of hospital discharges registered in hospitals in Spain (84.14% from public hospitals and 15.86% from private hospitals) [27]. The MBDS includes up to 14 discharge diagnoses and up to 20 procedures performed during the hospital stay. The MBDS provides encrypted patient identification numbers (the identification of individual patients is not possible in the MBDS), gender, date of birth, dates of hospital admission and discharge, medical institutions providing the services, the diagnosis and procedure codes according to the *International Classification of Diseases 9th Revision, Clinical Modification* (ICD-9-CM), as well as the outcome at discharge [28]. The Spanish MSSSI sets standards for record-keeping and performs periodic audits on the MBDS.

The data were treated with full confidentiality according to Spanish legislation. The MBDS is regulated by law explaining how institutions are required to utilize health-related personal data. The MSSSI of Spain confirmed that our study fulfilled all ethical considerations according to Spanish legislation. Thus, given the anonymous and mandatory nature of the data, informed consent was not required or necessary.

### Study variables

Sepsis was defined as the presence of an infection-associated diagnosis and organ dysfunction according to the criteria of Angus et al. [4] and adapted by other authors. We selected all acute-care hospitalizations with ICD-9-CM codes for both bacterial or fungal infections (ICD-9-CM codes used by Angus et al. [4]; see Additional file 1: Table S1) and a diagnosis of acute organ dysfunction (ICD-9-CM codes used by Angus et al. [4], Dombrovskiy et al. [20], and Shen et al. [29]; see Additional file 1: Table S2).

The main study factor was time, which was divided into calendar years and into three calendar periods (2000–2004, 2005–2009, and 2010–2013) related to protocols from the Surviving Sepsis Campaign (http://www.survivingsepsis.org/). The first guideline of the Surviving Sepsis Campaign was published in 2004 [19] with the stated goal of reducing mortality from sepsis by 25% in five years.

The main outcomes were the onset of sepsis (hospital admission with infection plus organ dysfunction) and death in patients with sepsis. The minor outcomes were the length of hospital stay (LOHS) and the costs related to sepsis.

### Statistical analyses

The percentage of sepsis was estimated as the proportion of hospital admissions that were diagnosed cases of sepsis. The percentage of in-hospital sepsis-related deaths was defined as the proportion of overall in-hospital deaths that were of septic patients. The CFR was estimated as the proportion of hospitalized patients with sepsis that died. The incidence of sepsis was defined as number of events per 1000 persons in the population. The mortality of sepsis was defined as number of deaths per 10,000 persons in the population. The incidence and mortality were standardized by age by direct method using as population reference the whole population in Spain (National Statistics Institute; http://www.ine.es/). Thus, the number of events was used as numerator and the denominator was the number of persons at risk by age group. We also calculated the odds for in-hospital sepsis-related death according to calendar period by using logistic regression models, which were adjusted by age, sex, and Charlson co-morbidity index (CCI; see Additional file 1: Table S4) [30].

The LOHS was obtained as the difference in days between the date of hospital admission and date of discharge or death. The day of hospital admission was considered to be day 0. Discharge on the same day was considered to be a one day stay. Costs were calculated using Diagnosis-Related Groups (DRG), which represents a medical-economic entity concerning a set of diseases requiring analogous management resources [27]. DRG data were extracted from the MBDS. All costs shown are adjusted for the increment of inflation for the same period in Spain.

Temporal trends by calendar periods were evaluated using Poisson distribution or ANOVA as appropriate.

All analyses were performed using the R statistical package, version 3.2.2 (GNU General Public License) [31]. All tests were two-tailed, with *p*-values of < 0.05 considered statistically significant.

## Results

### Patients’ characteristics

Table 1 shows the epidemiological and clinical characteristics of patients with sepsis. On the whole, 2,646,445 patients had sepsis from 2000 to 2013 in Spain (Table 1A), of whom 485,685 died (18.4%) (Table 1B). Most of the patients were men with medical conditions and CCI above 2. The organs most commonly affected were of the respiratory and renal systems. The percentages that were men, and that had dysfunction and infection of the respiratory system decreased significantly from 2000–2004 to 2010–2013 (*p* < 0.001), while the age, CCI, dysfunction and infection of the renal system, and number and percentage of acute organ dysfunction increased significantly from 2000–2004 to 2010–2013 (*p* < 0.001) (Table 1 A & B).

**Table 1:** Epidemiological and clinical characteristics of patients with sepsis in Spain from 2000 to 2013

	Entire period	2000–2004	2005–2009	2010–2013
A) *Patients with sepsis*				
No. of patients	2,646,445	686,062	984,207	976,176
Gender (male)	1,695,167 (64.1%)	458,453 (66.82%)	637,428 (64.77%)	599,286 (61.39%)
Age (years)	69.7 (20.0)	68.2 (19.8)	69.4 (20.1)	71 (20)
Medical condition (vs. Surgical condition)	2,312,446 (87.4%)	600,138 (87.48%)	860,081 (87.39%)	852,227 (87.3%)
Charlson index	2.38 (2.15)	2.1 (2)	2.4 (2.1)	2.6 (2.3)
Number of acute organ dysfunction				
Average	1.3 (0.6)	1.2 (0.5)	1.2 (0.6)	1.3 (0.7)
1 Acute organ dysfunction	2,137,412 (80.76%)	587,681 (85.66%)	802,906 (81.58%)	746,555 (76.48%)
2 Acute organ dysfunction	378,431 (14.30%)	76,217 (11.11%)	135,002 (13.72%)	167,212 (17.13%)
> 2 Acute organ dysfunction	130,872 (4.95%)	22,164 (3.23%)	46,299 (4.7%)	62,409 (6.39%)
Acute organ dysfunction				
Cardiovascular	270,390 (10.2%)	59,983 (8.74%)	99,886 (10.15%)	110,521 (11.32%)
Hematologic	138,943 (5.3%)	27,850 (4.06%)	48,487 (4.93%)	62,606 (6.41%)
Hepatic	79,577 (3%)	20,643 (3.01%)	30,405 (3.09%)	28,529 (2.92%)
Metabolic	143,684 (5.4%)	24,273 (3.54%)	50,643 (5.15%)	68,768 (7.04%)
Neurologic	113,885 (4.3%)	31,578 (4.6%)	40,561 (4.12%)	41,746 (4.28%)
Renal	658,149 (24.9%)	127,662 (18.61%)	221,451 (22.5%)	309,036 (31.66%)
Respiratory	1,926,562 (72.8%)	520,799 (75.91%)	735,912 (74.77%)	669,851 (68.62%)
Site of infection				
Central nervous system	19,342 (0.7%)	5716 (0.83%)	7387 (0.75%)	6239 (0.64%)
Circulatory	11,560 (0.4%)	2812 (0.41%)	4229 (0.43%)	4519 (0.46%)
Digestive	238,578 (9%)	59,591 (8.69%)	86,877 (8.83%)	92,110 (9.44%)
Genitourinary	506,124 (19.1%)	102,125 (14.89%)	175,486 (17.83%)	228,513 (23.41%)
Respiratory	1,583,486 (59.8%)	469,245 (68.4%)	594,592 (60.41%)	519,649 (53.23%)
B) *Sepsis-related deaths*				
No. of patients	485,685	130,927	181,008	173,750
Gender (male)	297,245 (61.2%)	82,713 (63.17%)	111,552 (61.63%)	102,980 (59.27%)
Age (years)	74.19 (15.95)	72.1 (16.6)	74 (16.1)	75.9 (15.1)
Medical condition (vs. Surgical condition)	381,972 (78.6%)	100,069 (76.43%)	141,779 (78.33%)	140,124 (80.65%)
Charlson index	2.92 (2.57)	2.6 (2.4)	2.9 (2.6)	3.1 (2.7)
Number of acute organ dysfunction				
Average	1.64 (0.91)	1.5 (0.8)	1.6 (0.9)	1.7 (1)
1 Acute organ dysfunction	283,418 (58.4%)	83,968 (64.13%)	106,277 (58.71%)	93,173 (53.62%)
2 Acute organ dysfunction	125,631 (25.9%)	31,803 (24.29%)	46,427 (25.65%)	47,401 (27.28%)
> 2 Acute organ dysfunction	76,636 (15.8%)	15,156 (11.58%)	28,304 (15.64%)	33,176 (19.09%)
Acute organ dysfunction				
Cardiovascular	135,996 (28%)	35,533 (27.14%)	50,943 (28.14%)	49,520 (28.5%)
Hematologic	36,600 (7.5%)	8115 (6.2%)	13,189 (7.29%)	15,296 (8.8%)
Hepatic	27,507 (5.7%)	6148 (4.7%)	10,676 (5.9%)	10,683 (6.15%)
Metabolic	39,646 (8.2%)	6827 (5.21%)	14,196 (7.84%)	18,623 (10.72%)
Neurologic	26,414 (5.4%)	7104 (5.43%)	9528 (5.26%)	9782 (5.63%)
Renal	179,575 (37%)	39,769 (30.37%)	64,127 (35.43%)	75,679 (43.56%)
Respiratory	350,234 (72.1%)	94,372 (72.08%)	132,677 (73.3%)	123,185 (70.9%)
Site of infection				
Central nervous system	4809 (1%)	1580 (1.21%)	1814 (1%)	1415 (0.81%)
Circulatory	4376 (0.9%)	1120 (0.86%)	1608 (0.89%)	1648 (0.95%)
Digestive	67,565 (13.9%)	18,937 (14.46%)	25,380 (14.02%)	23,248 (13.38%)
Genitourinary	91,702 (18.9%)	19,976 (15.26%)	33,065 (18.27%)	38,661 (22.25%)
Respiratory	252,718 (52%)	75,801 (57.9%)	95,289 (52.64%)	81,628 (46.98%)

Values are expressed as absolute number (percentage) and mean (standard deviation)

### Frequency of sepsis and sepsis-related death

**Fig. 1:**
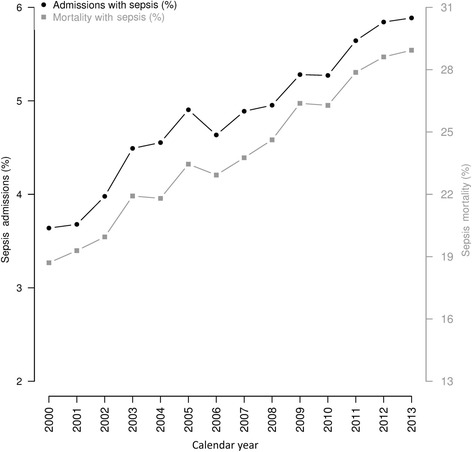
Trends of percentage of patients with sepsis and percentage of sepsis-related deaths among all hospital admissions and deaths

The percentage of hospital admissions due to sepsis increased from 3.6% in 2000 to 5.8% in 2013 (Fig. 1). The percentage of in-hospital sepsis-related deaths also increased, from 18.7% in 2000 to 29% in 2013 (Fig. 1).

### Sepsis-related rates

**Fig. 2:**
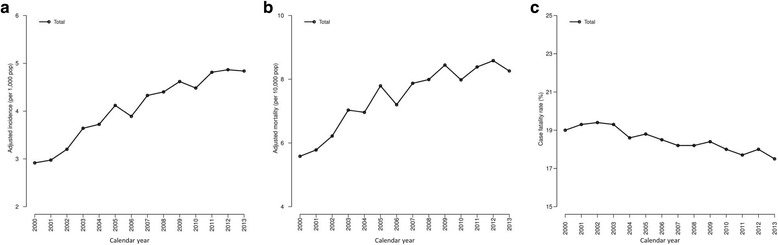
Trends of sepsis incidence, sepsis-related mortality, and case fatality rate in Spain from 2000 to 2013. **a** Population-adjusted incidence of sepsis; **b** Population-adjusted mortality related to sepsis; **c** Case fatality rate or proportion of deaths among patients hospitalized with sepsis

The incidence of sepsis (events per 1000 population) increased from 2.9 in 2000 to 4.8 in 2013 (*p* < 0.001) (Fig. 2a). When the follow-up was stratified by calendar periods, a significant upward trend was observed from 3.30 (2000–2004) to 4.28 (2005–2009) to 4.45 (2010–2013) (*p* < 0.001). Mortality due to sepsis (deaths per 10,000 population) increased from 5.6 in 2000 to 8.3 in 2013 (*p* < 0.001) (Fig. 2b). When stratified by calendar periods, a significant upward trend was observed from 6.34 (2000–2004) to 7.88 (2005–2009) to 7.89 (2010–2013) (*p* < 0.001). Moreover, the CFR (proportion of patients with sepsis who died) decreased from 19.0% in 2000 to 17.5% in 2013 (p < 0.001) (Fig. 2c). When stratified by calendar periods, a significant downward trend in CFR was observed from 19.1% (2000–2004) to 18.4% (2005–2009) to 17.9% (2010–2013) (*p* < 0.001). Additionally, the likelihood of death when compared to 2000–2003 decreased in 2004–2009 [adjusted odds ratio (aOR) of 0.90 (95% confidence interval (95% CI): 0.89, 0.91)] and 2010–2013 [aOR = 0.81 (95% CI: 0.80, 0.82)].

### National age-specific rates

**Fig. 3:**
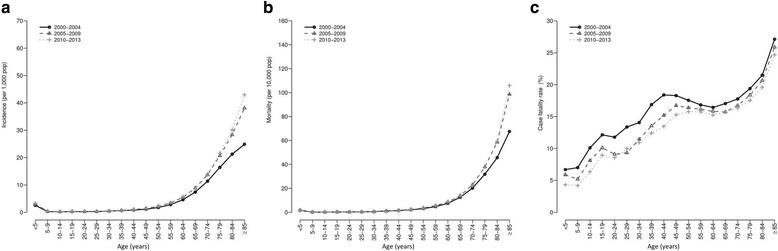
National age-specific rates (incidence, mortality, and case fatality rate) for sepsis in Spain (2000–2013) stratified by calendar periods. **a** incidence; **b** sepsis-related mortality; **c** case fatality rate or proportion of deaths among patients hospitalized with sepsis

The incidence and mortality of sepsis increased in late adulthood (aged 50–59 years), increased sharply in the elderly (aged > 65 years), and reach the highest values in the elderly over 85 (Fig. 3a–b). The trends in incidence and mortality were different according to calendar periods after age 65 with the highest values, although quite similar, in 2005–2009 and 2010–2013 (Fig. 3a–b). Moreover, the CFR increased from 7.2% in children to values above 20% in patients aged 45–49 years, but a break in the upward trend was found between ages 45–49 years and 60–64 years, since the values decreased or remained constant. Above age 65, the CFR increased quickly to values close to 30% by age 85 (Fig. 3c). The lowest values were found in 2005–2009 and 2010–2013 for all age groups, with very similar values (Fig. 3c).

### Hospital resource expenditure related to sepsis

**Fig. 4:**
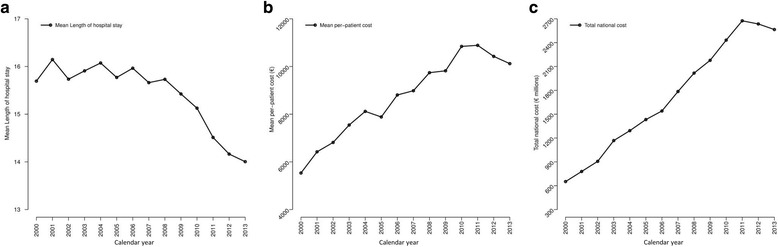
Evolution of cost and length of hospital stay for patients with sepsis in Spain from 2000 to 2013. **a** Average length of hospital stay (LOHS) per patient; **b** average cost per patient; **c** average total cost

The average LOHS was 15.3 days during the whole study period. The LOS values were lower in survivors than in non-survivors (15.1 vs. 16.4; *p* < 0.001), in medical condition than in surgical condition (12.2 vs. 37.9; *p* < 0.001), and in patients with organ dysfunction < 2 than in patients with organ dysfunction ≥2 (14.0 vs. 21.1; *p* < 0.001). Furthermore, the LOHS decreased from 15.7 in 2000 to 14.0 in 2013, particularly after 2008 (Fig. 4a). When the follow-up was stratified by calendar periods, a significant downward trend in LOHS was observed from 15.9 (2000–2004) to 15.7 (2005–2009) to 14.5 (2010–2013) (*p* < 0.001).

The average hospital cost per patient was 9090€ during whole study period. The average cost was lower in survivors than in non-survivors (8423€ vs. 10,219€; *p* < 0.001), in medical condition than in surgical condition (5334€ vs. 32,854€; *p* < 0.001), and in patients with organ dysfunction < 2 than in patients with organ dysfunction ≥2 (7307€ vs. 15,120€; *p* < 0.001). Furthermore, the average hospital cost per patient increased from 5533€ in 2000 to above 10,000€ after 2010, but then decreased after 2011 (Fig. 4b). When stratified by calendar periods, a significant upward trend in average cost was observed from 6991€ (2000–2004) to 9096€ (2005–2009) to 10,029€ (2010–2013) (*p* < 0.001). Moreover, the total national annual cost of hospitalization due to sepsis increased from 652 M euros in 2000 to over 2500 M after 2010, but with a decrease after 2011 (Fig. 4c).

### National age-specific hospital resource expenditure related to sepsis

**Fig. 5:**
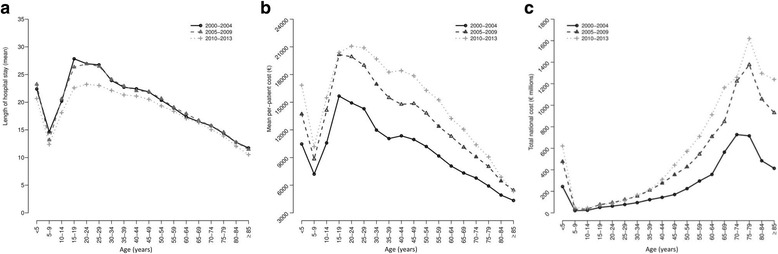
Age-specific hospital resource expenditure in Spain from 2000 to 2013 stratified by calendar periods. **a** average length of hospital stay (LOHS) per patient; **b** average cost per patient; **c** average total cost

The LOHS was high in infants and children under age 5 (LOHS 21.8), decreased quickly in slightly older children (age 5–9 years; LOHS 13.2), increased sharply for patients in early adulthood (age 20–24 yrs.; LOHS 25.3), and decreased sharply for the elderly (age 85 years; LOHS 11.1) (Fig. 5a). The lowest LOHS values were found in 2010–2013, with a clear decrease in LOHS for patients between age 15 and 54 (Fig. 5a).

The average hospital cost per patient was high in infants and children under age 5, decreased quickly in children age 5–9 years, increased sharply for patients in early adulthood (age 20–24 years), and decreased sharply for the elderly over 85 (Fig. 5b). By calendar periods, 2005–2009 marked a significant increase in spending, which was surpassed in 2010–2013 for patients between age 24 and 79 (Fig. 5b).

The total national cost was high in infants and children under 5, decreased quickly in children age 5–9 years, increased gradually until elderly patients age 70–79 years, and decreased sharply in the elderly over 80 (Fig. 5c). By calendar periods, the highest values were found in the last two calendar periods (2005–2009 and 2010–2013), especially in patients over 50 during the period 2010–2013 (Fig. 5c).

### Regional differences of sepsis epidemiology in Spain

Values of sepsis incidence and sepsis-related mortality were not uniform throughout the various regions (autonomous communities) of Spain (*p* < 0.001; Table 2). When the follow-up was stratified by calendar periods (Table 2), values of sepsis incidence increased significantly in all regions except Cantabria, and values of mortality increased significantly in all regions except Cantabria, Ceuta, and La Rioja (Table 2).

**Table 2:** Trends of sepsis incidence and sepsis-related mortality across Spanish regions (autonomous communities) from 2000 to 2013

	Incidence (events per 1000 population)				Mortality (deaths per 10,000 population)			
Regions	2000–2004	2005–2009	2010–2013	*p*-value	2000–2004	2005–2009	2010–2013	*p*-value
Andalusia	2.53 (2.52; 2.55)	3.47 (3.45; 3.49)	3.42 (3.4; 3.43)	**< 0.001**	5.8 (5.72; 5.88)	7.75 (7.67; 7.84)	7.89 (7.81; 7.98)	**< 0.001**
Aragon	4 (3.95; 4.05)	4.76 (4.71; 4.82)	4.67 (4.62; 4.73)	**< 0.001**	9.39 (9.14; 9.63)	10.46 (10.21; 10.71)	10.49 (10.25; 10.74)	**< 0.001**
Asturias	3.95 (3.9; 4.01)	4.77 (4.71; 4.83)	5.44 (5.38; 5.5)	**< 0.001**	7.1 (6.87; 7.32)	8.33 (8.09; 8.58)	10.27 (9.99; 10.54)	**< 0.001**
Balearic Islands	3.21 (3.16; 3.26)	4.38 (4.33; 4.44)	4.52 (4.46; 4.58)	**< 0.001**	5.49 (5.27; 5.7)	6.63 (6.41; 6.86)	6.6 (6.39; 6.82)	**< 0.001**
Basque country	3.26 (3.23; 3.3)	4.67 (4.62; 4.71)	5.09 (5.05; 5.13)	**< 0.001**	5.54 (5.4; 5.68)	7.13 (6.97; 7.29)	7.94 (7.78; 8.11)	**< 0.001**
Canary Islands	1.35 (1.33; 1.37)	2.07 (2.04; 2.09)	2.21 (2.19; 2.24)	**< 0.001**	3.34 (3.22; 3.46)	4.94 (4.8; 5.08)	4.97 (4.83; 5.1)	**< 0.001**
Cantabria	4.13 (4.06; 4.21)	4.18 (4.1; 4.25)	4.16 (4.09; 4.23)	0.616	8.22 (7.87; 8.56)	9.32 (8.97; 9.68)	8.65 (8.32; 8.99)	0.096
Castile-La Mancha	4.13 (4.09; 4.18)	5.5 (5.45; 5.55)	5.66 (5.62; 5.71)	**< 0.001**	6.32 (6.15; 6.48)	8.97 (8.79; 9.16)	9.84 (9.64; 10.03)	**< 0.001**
Castille and Leon	4.22 (4.19; 4.26)	6.01 (5.97; 6.05)	6.36 (6.32; 6.41)	**< 0.001**	8.63 (8.47; 8.8)	11.6 (11.41; 11.79)	12.05 (11.86; 12.24)	**< 0.001**
Catalonia	3.86 (3.84; 3.88)	5.14 (5.12; 5.17)	5.59 (5.56; 5.61)	**< 0.001**	6.31 (6.22; 6.4)	8 (7.91; 8.1)	8.11 (8.02; 8.21)	**< 0.001**
Ceuta	2.06 (1.91; 2.2)	3.47 (3.28; 3.66)	2.63 (2.47; 2.79)	**< 0.001**	5.32 (4.56; 6.08)	6.76 (5.91; 7.61)	6.21 (5.43; 7)	0.132
Extremadura	3.03 (2.98; 3.08)	3.9 (3.85; 3.96)	4.66 (4.6; 4.72)	**< 0.001**	5.64 (5.44; 5.84)	8.17 (7.93; 8.41)	9.09 (8.83; 9.34)	**< 0.001**
Galicia	4.4 (4.37; 4.44)	5.55 (5.51; 5.59)	5.87 (5.83; 5.91)	**< 0.001**	8.36 (8.2; 8.51)	10.45 (10.28; 10.62)	10.88 (10.71; 11.06)	**< 0.001**
La Rioja	4.38 (4.27; 4.49)	4.85 (4.74; 4.96)	4.62 (4.52; 4.73)	**0.003**	6.47 (6.05; 6.9)	6.23 (5.83; 6.62)	6.65 (6.25; 7.06)	0.514
Madrid	3.86 (3.84; 3.89)	5.06 (5.04; 5.09)	5.67 (5.64; 5.7)	**< 0.001**	7.17 (7.07; 7.27)	8.57 (8.47; 8.68)	8.94 (8.84; 9.05)	**< 0.001**
Murcia	2.75 (2.71; 2.79)	3.35 (3.3; 3.39)	3.77 (3.73; 3.82)	**< 0.001**	5.61 (5.42; 5.79)	6.6 (6.41; 6.79)	7.14 (6.95; 7.33)	**< 0.001**
Melilla	2.59 (2.42; 2.77)	3.13 (2.94; 3.32)	3.07 (2.9; 3.25)	**< 0.001**	5.27 (4.49; 6.05)	8.6 (7.61; 9.58)	8.75 (7.81; 9.69)	**< 0.001**
Navarre	2.78 (2.72; 2.84)	3.93 (3.86; 4)	3.72 (3.65; 3.79)	**< 0.001**	4.51 (4.26; 4.76)	7.15 (6.85; 7.46)	6.84 (6.55; 7.13)	**< 0.001**
Valencian community	2.28 (2.26; 2.3)	3.23 (3.2; 3.25)	3.79 (3.76; 3.81)	**< 0.001**	4.74 (4.64; 4.83)	6.49 (6.39; 6.6)	7.39 (7.28; 7.5)	**< 0.001**
p-value	**< 0.001**	**< 0.001**	**< 0.001**		**< 0.001**	**< 0.001**	**< 0.001**	

Values were expressed as rate (95% CI)

## Discussion

Our research shows the growing burden of sepsis during the early twenty-first century in Spain. In this study, the percentage of sepsis cases and in-hospital sepsis-related deaths, with respect to overall hospital admissions and deaths, increased from 2000 to 2013. The adjusted rates of incidence and mortality also increased, and although their values were not uniform across regions in Spain, these rate values increased significantly in most regions when the follow-up was stratified by calendar periods. Prior studies have also demonstrated this trend, regardless of the algorithm used to determine the diagnosis of sepsis from the ICD-9 codes [4, 11, 20, 21, 24, 29]. Additionally, in our findings, sepsis and sepsis-related deaths occurred more frequently in older people, who also had more comorbidity and developed a higher rate of acute organ failure, likely due to the natural process of aging and an accompanying increase in disease severity. These trends may be due to the increasing age of the Spanish population, greater comorbidity, greater use of invasive procedures and immunosuppressive drugs, and nosocomial infections generally associated with resistant microorganisms [2, 6]. However, we should not exclude a possible bias due to a greater awareness of the severity of sepsis [18]. Furthermore, the introduction and improvement of the management of ICD-9 codes may have facilitated coding in medical records similar to MBDS [7]. Namely, as awareness of sepsis has increased during the last decade, the coding practices might have been become more inclusive [32]. Thereby, if an increasing number of less sick patients were included as patients with sepsis, the incidence of sepsis could have increased. However, we have not been able to evaluate this hypothesis via the MBDS and we do not have access to severity of illness scores such as SOFA or APACHE for adjusting the analysis.

We found that the CFR of sepsis decreased during the 14-year study period, consistent with the overall decreasing trend observed in prior studies [33]. Although small in percentage, the decline in CFR may be considered remarkable given the increases in age, morbidity, and severity of illness. In addition to the above-mentioned comments, this downward trend may also be attributable to general improvements in intensive care and the Surviving Sepsis Campaign [19] that have resulted in an increased awareness of sepsis over time. However, we should not exclude possible bias due to a greater awareness of the severity of sepsis [18], as mentioned above. As with other metrics, if an increasing number of less sick patients were diagnosed with sepsis, the CFR could decrease.

Overall, the variations in incidence, mortality, and CFR in our study seem to be lower in comparison to prior studies [4, 11, 20, 21, 24, 29, 33]. In addition, incidence, mortality, and CFR changes are diminished in the last calendar period (2010–2013), which could indicate that they are reaching a plateau. In fact, regardless of statistical significance, the differences between the last two calendar periods (2004–2009 vs. 2010–2013) are very low and virtually nonexistent. This slowdown was also observed when incidence, mortality, and CFR were analyzed by age strata. The highest values were found in elderly over 65, especially in patients over 85, but these rates were quite similar between 2005 and 2009 and 2010–2013 within each age group.

The average LOHS per calendar year or period is useful from the point of view of costs. The reduction in LOHS implies a faster recovery and a reduction of costs and hospital resources. However, patients may also be discharged with high degrees of disability, and perhaps, not even going home but to a nursing facility. Previous studies have showed values of LOHS between 17 and 30 days before 2000, and 9–15 days after 2000 [4, 11, 21, 22, 34]. In our study, LOHS values were close to 16 days during the first years of the century, but following 2008 decreased to about 14 by 2013. The LOHS trend was not consistent with the trend of CFR during the study period, since the LOHS values showed a flat trend in the first two calendar periods (2000–2004 and 2005–2009) and decreased in 2010–2013; whereas the CFR values showed a smooth downward trend during the entire study period (2000–2013). In addition, the trend of LOHS was also not consistent with the increase of hospital costs (per patient and total). However, we must also highlight that the decreasing trend in LOHS is particularly important in the context of higher age, comorbidities, and organ failures in patients. This decrease in costs that is observed after 2011 could be due to the economic crisis [15–17], but nor should we rule out the impact of other factors such as greater adherence to treatment guidelines [25]. For example, educational clinical initiatives promoting best practices in the management of sepsis have been developed in recent years in Spain [25].

When analysis by age strata was carried out, the highest values of LOHS and hospital cost per patient were found in children (< 5 years), teenagers (15–19 years) and adults (aged 20–59 years). Thus, the trends of LOHS and hospital cost per patient were quite similar, and patients with higher LOHS accounted for more hospital spending. On aggregate, the highest values of total national cost were found in elderly patients (aged 70–79 years), because they were the most numerous sepsis patients. It is also important to note that the impact of calendar periods on values of LOHS was only evident in the last period (2010–2013), mainly in patients ranging from 15 to 35 years in age, since the first two periods had very similar values; while the hospital costs (per patient and total) reached a substantial increase in 2005–2009, and was even higher in 2010–2013.

One of the strengths of this study is that it analyzed nationwide data. Our study captures acute-care hospitalizations for sepsis in Spain via MBDS and ICD-9 codes, which is well-established in sepsis epidemiology for assessing its trends and the need for preventive and therapeutic care and for service planning [22, 33]. Our study was performed according to a similar criterion as what was used by Angus et al. [4], and was only modified to update the codes of acute organ dysfunction with ICD-9-CM codes used by Dombrovskiy et al. [20] and Shen et al. [29]. The Angus criteria [4] is one of the most well-known and highly cited implementations of an ICD-coded case definition for sepsis [35]. Indeed, it is important to also note that the criteria of Angus et al. [4] coincides with the current definition of sepsis [1]. In our study, our figures are similar to data reported in other articles that utilize the “Angus” algorithm [4, 29, 33]. A recent study has compared the “Angus” algorithm to the “Martin” algorithm [36], which selected sepsis cases based on the presence of an ICD-9-CM code for infection and acute organ dysfunction. The “Angus” approach has a moderate to low sensitivity of 50.3% and a positive predictive value of 70.7%, whereas the “Martin” algorithm has a very low sensitivity of 16.8% but a high positive predictive value of 97.6% [36]. Thus, the “Angus” algorithm may capture more sepsis cases than the “Martin” algorithm, and even so, it is still underestimating the number of cases [36]. Additionally, the mortality trends identified using administrative data seem to be similar to those identified in clinical trial participants, and support the use of ICD-9 data, integrated into the “Angus” algorithm, to monitor mortality trends in patients with sepsis [33].

We should note the existence of a paper on a very similar topic using the same dataset and another similar criterion of Angus et al. [24]. Incidence and mortality rate values in our article were higher than in the Bouza et al. article, whereas CFR values were higher in their article [24]. However, the trend of incidence, mortality, and CFR were similar in both studies. Moreover, our analysis covers a broader period of time (2000–2013), which includes the years in which the impact of the economic crisis was stronger, as well as an analysis of hospital resource expenditures and associated costs related to sepsis. These two details result in different findings and conclusions than those described in the Bouza’s article [24].

We must also acknowledge other possible limitations of our study. Firstly, this study was retrospective using administrative databases, and thus, the acquisition of some clinical data (community acquired or nosocomial nature of sepsis, or prognostic scores such as Child-Pugh, MELD, SOFA, or CLIF-SOFA) was unavailable from the MBDS records. Furthermore, we do not know the reason for admission of these patients and if these patients were admitted due to sepsis or acquired sepsis in the hospital prior to death. Secondly, due to the use of the administrative databases, the inaccuracy in differentiating the etiology of the diseases and the reporting of organ dysfunction could have caused a confusion bias. For example, we found an unexpected high incidence of urinary infection and low incidence of cardiovascular dysfunction, possibly due to a bias in diagnosis reporting due to the fact that it may be more reliable to report urinary infection than cardiovascular dysfunction versus other sources. In this context, grouping of ICD-9-CM codes into comorbidities, organ dysfunction, and site of infection (Additional file 1: Tables S1-S4) may have been the best approach to solve this issue, considering that we have not used the ICD-9 code 995.9× (sepsis or severe sepsis) nor 785.52 (septic shock) due to these codes being highly problematic. Furthermore, we did not have data of the potential accuracy of the Spanish MBDS for sepsis-related diagnoses, which could be a significant limitation. Thirdly, MBDS data are anonymous, and it is impossible to identify whether a patient has been hospitalized more than once in different hospitals. This may have caused a slight overestimation of incidence and mortality rates since around one-third of the patients surviving their first episode of sepsis may develop other subsequent episodes [29]. Fourthly, the DRG system was the only viable method to calculate sepsis costs via the MBDS. DRGs may not be a precise method for determining costs, particularly in ICU patients, because different conditions in a DRG may have widely varying costs and different levels of intensity of care cannot (without adjustment) be distinguished within a DRG. However, the DRG system is readily available and provides a uniform methodology to get a common currency of hospital activity, which might be applied to all hospitals of a National Health System. Fifthly, in regard to the referral population, the population in Spain may vary as to the number of foreign habitants that may develop sepsis and be treated in a public hospital. Besides, private hospitals may also attend a larger proportion of septic patients. However, we did not have data of these two variables and they could not be considered for the analysis.

## Conclusions

Our data show that sepsis has been an increasing burden (hospital admission, deaths, and costs) in the Spanish public health system during the early twenty-first century (2000–2013), but incidence and mortality seem to have stabilized in the last calendar period (2010–2013). Moreover, there was also a significant decrease in LOHS values in 2010–2013, accompanied by a decrease in the hospital costs per patient and total national costs for sepsis after 2011. These conclusions would benefit from further attempts to corroborate these findings.

## Additional file

Additional file 1: SUPPLEMENTARY DIGITAL CONTENT (**Tables S1-S4**). **Table S1**. International Classification of Diseases, 9th Revision, Clinical Modification (ICD-9-CM) codes for bacterial and fungal infections. **Table S2**. International Classification of Diseases, 9th Revision, Clinical Modification (ICD-9-CM) codes for acute organ dysfunction. **Table S3**. International Classification of Diseases, 9th Revision, Clinical Modification (ICD-9-CM) codes used to identify the source of infection causing sepsis. **Table S4**. International Classification of Diseases, 9th Revision, Clinical Modification (ICD-9-CM) coding algorithms for Charlson comorbidities. (DOCX 51 kb)

## Abbreviations

AOR: Adjusted odds ratio
CCI: Charlson co-morbidity index
CFR: Case fatality rate
DRG: Diagnosis-Related Groups
ICD-9-CM: International Classification of Diseases 9th Revision, Clinical Modification
ICU: intensive care unit
LOHS: Length of hospital stay
MBDS: Minimum Basic Data Set
MSSSI: Ministry of Health Social Services and Equality
SDC: Supplementary Digital Content
