# Supplementary material for: Epidemiological trends of sepsis in the twenty-first century (2000–2013): an analysis of incidence, mortality, and associated costs in Spain
Source: Popul Health Metr. 2018 Feb 12;16:4. doi: 10.1186/s12963-018-0160-x (PMC5809921; doi:10.1186/s12963-018-0160-x)
Supplement: Supplementary file 1 — SUPPLEMENTARY DIGITAL CONTENT (Tables S1-S4). Table S1. International Classification of Diseases, 9th Revision, Clinical Modification (ICD-9-CM) codes for bacterial and fungal infections. Table S2. International Classification of Diseases, 9th Revision, Clinical Modification (ICD-9-CM) codes for acute organ dysfunction. Table S3. International Classification of Diseases, 9th Revision, Clinical Modification (ICD-9-CM) codes used to identify the source of infection causing sepsis. Table S4. International Classification of Diseases, 9th Revision, Clinical Modification (ICD-9-CM) coding algorithms for Charlson comorbidities. (DOCX 51 kb) [file 12963_2018_160_MOESM1_ESM.docx]

**Table S1.** *International Classification of Diseases, 9th Revision, Clinical Modification* (ICD-9-CM) codes for bacterial and fungal infections. Adapted from Angus et al. ([1](#_ENREF_1)).

| **ICD-9-CM Code** | **ICD-9-CM Code Description** |
| --- | --- |
| 001 | Cholera |
| 002 | Typhoid/paratyphoid fever |
| 003 | Other salmonella infection |
| 004 | Shigellosis |
| 005 | Other food poisoning |
| 008.0 | Intestinal infections due to *Escherichia coli* |
| 008.1 | Intestinal infections due to Arizona group of paracolon bacill |
| 008.2 | Intestinal infections due to *Aerobacter aerogenes* |
| 008.3 | Intestinal infections due to *Proteus (mirabilis) (morganii)* |
| 008.4 | Intestinal infections due to unspecified bacteria |
| 008.5 | Bacterial enteritis, unspecified |
| 009 | Ill-defined intestinal infection |
| 013 | Central nervous system tuberculosis |
| 018 | Miliary tuberculosis |
| 020 | Plague |
| 021 | Tularemia |
| 022 | Anthrax |
| 023 | Brucellosis |
| 024 | Glanders |
| 025 | Melioidosis |
| 026 | Rat-bite fever |
| 027 | Other bacterial zoonoses |
| 032 | Diphtheria |
| 033 | Whooping cough |
| 034 | Streptococcal throat/scarlet fever |
| 035 | Erysipelas |
| 036 | Meningococcal infection |
| 037 | Tetanus |
| 038 | Septicemia |
| 039 | Actinomycotic infections |
| 040 | Other bacterial diseases |
| 041 | Bacterial infection in other diseases not specified |
| 098 | Gonococcal infections |
| 100 | Leptospirosis |
| 101 | Vincent’s angina |
| 112.0 | Candidiasis, of mouth |
| 112.4 | Candidiasis, of lung |
| 112.5 | Candidiasis, disseminated |
| 112.8 | Candidiasis, of other specified sites |
| 114 | Coccidioidomycosis |
| 115 | Histoplasmosis |
| 116 | Blastomycotic infection |
| 117 | Other mycoses |
| 118 | Opportunistic mycoses |
| 320 | Bacterial meningitis |
| 321.0 | Cryptococcal meningitis |
| 321.1 | Meningitis in other fungal diseases |
| 324 | Central nervous system abcess |
| 325 | Phlebitis of intracranial sinus |
| 360.0 | Purulent endophthalmitis |
| 376.0 | Acute inflammation of orbit |
| 380.14 | Malignant otitis externa |
| 383.0 | Acute mastoiditis |
| 420.99 | Acute pericarditis due to other specified organisms |
| 421 | Acute or subacute endocarditis |
| 461 | Acute sinusitis |
| 462 | Acute pharyngitis |
| 463 | Acute tonsillitis |
| 464 | Acute laryngitis/tracheitis |
| 465 | Acute upper respiratory infection of multiple sites/not otherwise specified |
| 475 | Peritonsillar abscess |
| 481 | Pneumococcal pneumonia |
| 482 | Other bacterial pneumonia |
| 485 | Bronchopneumonia with organism not otherwise specified |
| 486 | Pneumonia, organism not otherwise specified |
| 491.21 | Acute exacerbation of obstructive chronic bronchitis |
| 494 | Bronchiectasis |
| 510 | Empyema |
| 513 | Abscess of lung and mediastinum |
| 522.5 | Periapical abscess without sinus |
| 522.7 | Periapical abscess with sinus |
| 526.4 | Inflammatory conditions of the jaw |
| 527.3 | Abscess of the salivary glands |
| 528.3 | Cellulitis and abscess of oral soft tissue |
| 540 | Acute appendicitis |
| 541 | Appendicitis not otherwise specified |
| 542 | Other appendicitis |
| 562.01 | Diverticulitis of the small intestine without hemorrhage |
| 562.03 | Diverticulitis of the small intestine with hemorrhage |
| 562.11 | Diverticulitis of colon without hemorrhage |
| 562.13 | Diverticulitis of colon with hemorrhage |
| 566 | Abscess of the anal and rectal regions |
| 567 | Peritonitis |
| 569.5 | Intestinal abscess |
| 569.61 | Infection of colostomy or enterostomy |
| 569.83 | Perforation of intestine |
| 572.0 | Abscess of liver |
| 572.1 | Portal pyemia |
| 575.0 | Acute cholecystitis |
| 590 | Kidney infection |
| 599.0 | Urinary tract infection not otherwise specified |
| 601 | Prostatic inflammation |
| 604 | Orchitis and epididymitis |
| 614 | Female pelvic inflammation disease |
| 615 | Uterine inflammatory disease |
| 616.3 | Abcess of Bartholin’s gland |
| 616.4 | Other abcess of vulva |
| 634.0 | Spontaneous abortion, complicated by genital tract and pelvic infection |
| 635.0 | Legally induced abortion, complicated by genital tract and pevic infection |
| 636.0 | Illegally induced abortion, complicated by genital tract and pelvic infection |
| 637.0 | Unspecified abortion, complicated by genital tract and pelvic infection |
| 638.0 | Failed attempted abortion, complicated by genital tract and pelvic infection |
| 639.0 | Complications following abortion and ectopic and molar pregnancies,genital and pelvic infection |
| 646.6 | Infections of genitourinary tract in pregnancy |
| 658.4 | Infection of amniotic cavity |
| 670 | Major puerperal infection |
| 675.1 | Abscess of breast |
| 681 | Cellulitis, finger/toe |
| 682 | Other cellulitis or abscess |
| 683 | Acute lymphadenitis |
| 685.0 | Pilonidal cyst, with abscess |
| 686 | Other local skin infection |
| 711.0 | Pyogenic arthritis |
| 728.86 | Necrotizing fasciitis |
| 730 | Osteomyelitis |
| 790.7 | Bacteremia |
| 958.3 | Posttraumatic wound infection, not elsewhere classified |
| 996.6 | Infection or inflammation of device/graft |
| 998.5 | Postoperative infection |
| 999.3 | Infectious complication of medical care not otherwise classified |

**Table S2.** *International Classification of Diseases, 9th Revision, Clinical Modification* (ICD-9-CM) codes for acute organ dysfunction. Adapted from Dombrovskiy et al. ([2](#_ENREF_2)), Shen et al. ([3](#_ENREF_3)), and Angus et al. ([1](#_ENREF_1)).

| **Organ System** | **ICD-9-CM Code** | **ICD-9-CM Code Description** |
| --- | --- | --- |
| Cardiovascular | 427.5 | Cardiac arrest |
|  | 458.0 | Orthostatic hypotension |
|  | 458.8 | Other specified hypotension |
|  | 458.9 | Hypotension, unspecified |
|  | 785.5 | Shock without mention of trauma |
|  | 796.3 | Hypotension, transient |
| Hematologic | 286.2 | Disseminated intravascular coagulation |
|  | 286.6 | Defibrination syndrome |
|  | 286.9 | Other and unspecified coagulation defects |
|  | 287.3-5 | Thrombocytopenia, primary, secondary or unspecified |
|  | 790.92 | Abnormal coagulation profile |
| Hepatic | 570 | Acute and subacute necrosis of liver |
|  | 572.2 | Hepatic encephalopathy |
|  | 573.3 | Hepatitis (septic & not elsewhere classified) |
|  | 573.4 | Hepatic infarction |
| Neurologic | 293 | Transient organic psychosis |
|  | 348.1 | Anoxic brain damage |
|  | 348.3 | Encephalopathy, acute |
|  | 780.01 | Coma |
|  | 780.09 | Altered consciousness, unspecified |
|  | 89.14 | Electroencephalography |
| Renal | 580.x | Acute glomerulonephritis |
|  | 584.x | Acute renal failure |
|  | 586 | Renal shutdown, renal failure unspecified |
|  | 39.95 | Hemodialysis |
| Respiratory | 518.5 | Pulmonary insufficiency following trauma and surgery |
|  | 518.8 | Respiratory failure |
|  | 786.03 | Apnea |
|  | 799.1 | Respiratory arrest |
|  | 786.09 | Respiratory insufficiency |
|  | 96.7 (96.71,96.72) | Ventilator management |
|  | 96.04 | Endotracheal intubation (emergency procedure) |
|  | 93.90 | Continuous positive airway pressureº |
| Metabolic | 276.2 | Acidosis, metabolic or lactic |

ICD-9-CM, International Classification of Diseases, Ninth Revision, Clinical Modification.

**Table S3.** *International Classification of Diseases, 9th Revision, Clinical Modification* (ICD-9-CM) codes used to identify the source of infection causing sepsis. Adapted from Esper et al. ([4](#_ENREF_4)) and Wang et al. ([5](#_ENREF_5)), and subsequently modified for HIV patients.

| **Organ System** | **ICD-9-CM Code** | **ICD-9-CM Code Description** |
| --- | --- | --- |
| **Nervous** |  |  |
|  | 013 | Tuberculosis of meninges and central nervous system |
|  | 036 | Meningococcal infection |
|  | 091.81 | Acute syphilitic meningitis (secondary) |
|  | 098.82 | Neurosyphilis |
|  | 320 | Bacterial meningitis |
|  | 321 | Cryptococcal meningitis |
|  | 321.1 | Meningitis in other fungal diseases |
|  | 324 | Central nervous system abscess |
|  | 325 | Phlebitis of intracranial sinus |
|  | 360 | Purulent endophthalmitis |
|  | 376 | Acute inflammation of orbit |
|  | 380.14 | Malignant otitis externa |
|  | 383 | Acute mastoiditis |
| **Circulatory** |  |  |
|  | 093 | Cardiovascular syphilis |
|  | 098.83 – 098.84 | Gonococcal infections |
|  | 036.4 | Meningococcal carditis |
|  | 391.2 | Acute rheumatic myocarditis |
|  | 420.99 | Acute pericarditis due to other specified organisms |
|  | 421 | Acute or subacute endocarditis |
| **Respiratory** |  |  |
|  | 010.1 | Tuberculous pleurisy in primary progressive tuberculosis |
|  | 011 | Pulmonary tuberculosis |
|  | 012 | Other respiratory tuberculosis |
|  | 018 | Miliary tuberculosis |
|  | 031.0 | Pulmonary diseases due to other mycobacteria |
|  | 032 | Diphtheria |
|  | 034 | Streptococcal throat/scarlet fever |
|  | 098.6 | Gonococcal infection of pharynx |
|  | 112.4 | Candidiasis, of lung |
|  | 114.0 | Primary coccidioidomycosis (pulmonary) |
|  | 114.2 | Primary extrapulmonary coccidioidomycosis |
|  | 115.15 | Histoplasma duboisii pneumonia |
|  | 115.05 | Histoplasma capsulatum pneumonia |
|  | 115.95 | Histoplasmosis pneumonia unspecified |
|  | 117.5 | Cryptococcus neoformans |
|  | 117.3 | Aspergillosis |
|  | 136.3 | Pneumocystosis |
|  | 461 | Acute sinusitis |
|  | 462 | Acute pharyngitis |
|  | 463 | Acute tonsillitis |
|  | 464 | Acute laryngitis/tracheitis |
|  | 465 | Acute upper respiratory infection of multiple sites/not |
|  |  | otherwise specified |
|  | 475 | Peritonsillar abscess |
|  | 480 | Viral pneumonia |
|  | 481 | Pneumococcal pneumonia |
|  | 482 | Other bacterial pneumonia |
|  | 483 | Pneumonia due to other specified organism |
|  | 485 | Bronchopneumonia with organism not otherwise specified |
|  | 486 | Pneumonia, organism not otherwise specified |
|  | 487.0 | Influenza |
|  | 491.21 | Acute exacerbation of obstructive chronic bronchitis |
|  | 494 | Bronchiectasis |
|  | 510 | Empyema |
|  | 513 | Abscess of lung and mediastinum |
| **Digestive** |  |  |
|  | 001 | Cholera |
|  | 002 | Typhoid/paratyphoid fever |
|  | 003 | Other salmonella infection |
|  | 004 | Shigellosis |
|  | 005 | Other food poisoning |
|  | 008 | Intestinal infections due to *Escherichia coli* |
|  | 008.1 | Intestinal infections due to Arizona group of paracolon bacillus |
|  | 008.2 | Intestinal infections due to *Aerobacter aerogenes* |
|  | 008.3 | Intestinal infections due to *Proteus* (*Mirabilis morganii*) |
|  | 008.4 | Intestinal infections due to unspecified bacteria |
|  | 008.5 | Bacterial enteritis, unspecified |
|  | 009 | Ill-defined intestinal infection |
|  | 014 | Tuberculosis of intestines peritoneum and mesenteric glands |
|  | 129 | Intestinal parasitism unspecified |
|  | 522.5 | Periapical abscess without sinus |
|  | 522.7 | Periapical abscess with sinus |
|  | 526.4 | Inflammatory conditions of the jaw |
|  | 527.3 | Abscess of the salivary glands |
|  | 528.3 | Cellulitis and abscess of oral soft tissue |
|  | 540 | Acute appendicitis |
|  | 541 | Appendicitis not otherwise specified |
|  | 542 | Other appendicitis |
|  | 562.01 | Diverticulitis of the small intestine without hemorrhage |
|  | 562.03 | Diverticulitis of the small intestine with hemorrhage |
|  | 562.11 | Diverticulitis of colon without hemorrhage |
|  | 562.13 | Diverticulitis of colon with hemorrhage |
|  | 566 | Abscess of the anal and rectal regions |
|  | 567 | Peritonitis |
|  | 569.5 | Intestinal abscess |
|  | 569.61 | Infection of colostomy or enterostomy |
|  | 569.83 | Perforation of intestine |
|  | 572 | Abscess of liver |
|  | 572.1 | Portal pyemia |
|  | 575 | Acute cholecystitis |
| **Genitourinary** |  |  |
|  | 016 | Tuberculosis of genitourinary system |
|  | 098.17 | Gonococcal salpingitis specified as acute |
|  | 112.2 | Candidiasis of other urogenital sites |
|  | 590 | Kidney infection |
|  | 599 | Urinary tract infection not otherwise specified |
|  | 601 | Prostatic inflammation |
|  | 604 | Orchitis and epididymitis |
|  | 614 | Female pelvic inflammation disease |
|  | 615 | Uterine inflammatory disease |
|  | 616.3 | Abscess of Bartholin’s gland |
|  | 616.4 | Other abscess of vulva |
| **Pregnancy** |  |  |
|  | 634 | Spontaneous abortion, complicated by genital tract and pelvic |
|  |  | infection |
|  | 635 | Legally induced abortion, complicated by genital tract and |
|  |  | pelvic infection |
|  | 636 | Illegally induced abortion, complicated by genital tract and |
|  |  | pelvic infection |
|  | 637 | Unspecified abortion, complicated by genital tract and pelvic |
|  |  | infection |
|  | 638 | Failed attempted abortion, complicated by genital tract and |
|  |  | pelvic infection |
|  | 639 | Complications following abortion and ectopic and molar |
|  |  | pregnancies |
|  | 646.6 | Infections of genitourinary tract in pregnancy |
|  | 658.4 | Infection of amniotic cavity |
|  | 670 | Major puerperal infection |
|  | 675.1 | Abscess of breast |
| **Skin, soft tissue, or bone** | |  |
|  | 003.24 | Salmonella osteomyelitis |
|  | 015 | Tuberculosis of bones and joints |
|  | 017 | Tuberculosis of other organs |
|  | 031.1 | Cutaneous diseases due to other mycobacteria |
|  | 035 | Erysipelas |
|  | 036.82 | Meningococcal arthropathy |
|  | 040.0 | Gas gangrene |
|  | 095.5 | Syphilis of bone |
|  | 098.5 | Gonococcal infection of joint |
|  | 681 | Cellulitis, finger/toe |
|  | 682 | Other cellulitis or abscess |
|  | 683 | Acute lymphadenitis |
|  | 685 | Pilonidal cyst, with abscess |
|  | 686 | Other local skin infection |
|  | 711 | Pyogenic arthritis |
|  | 728.86 | Necrotizing fasciitis |
|  | 730 | Osteomyelitis |
| **Other** |  |  |
|  | 790.7 | Bacteremia |
|  | 958.3 | Posttraumatic wound infection, not elsewhere classified |
|  | 996.6 | Infection or inflammation of device/graft |
|  | 998.5 | Postoperative infection |
|  | 999.3 | Infectious complication of medical care not otherwise classified |

**Table S4.** *International Classification of Diseases, 9th Revision, Clinical Modification* (ICD-9-CM) coding algorithms for Charlson comorbidities. Adapted from Quan et al. ([6](#_ENREF_6)).

| **Comorbidities** | **ICD-9-CM** |
| --- | --- |
| Myocardial infarction | 410.x, 412.x |
| Congestive heart failure | 428.x |
| Peripheral vascular disease | 443.9, 441.x, 785.4, V43.4, Procedure 38.48 |
| Cerebrovascular disease | 430.x–438.x |
| Dementia | 290.x |
| Chronic pulmonary disease | 490.x–505.x, 506.4 |
| Rheumatic disease | 710.0, 710.1, 710.4, 714.0–714.2, 714.81, 725.x |
| Peptic ulcer disease | 531.x–534.x |
| Mild liver disease | 571.2, 571.4–571.6 |
| Diabetes without chronic complication | 250.0–250.3, 250.7 |
| Diabetes with chronic complication | 250.4–250.6 |
| Hemiplegia or paraplegia | 344.1, 342.x |
| Renal disease | 582.x, 583–583.7, 585.x, 586.x, 588.x |
| Any malignancy, including lymphoma and leukemia, except malignant neoplasm of skin | 140.x–172.x, 174.x.–195.8, 200.x–208.x |
| Moderate or severe liver disease | 456.0–456.21, 572.2–572.8 |
| Metastatic solid tumor | 196.x–199.1 |
| AIDS/HIV | 042.x–044.x |

**REFERENCES**

1. Angus DC, Linde-Zwirble WT, Lidicker J, Clermont G, Carcillo J, Pinsky MR. Epidemiology of severe sepsis in the United States: analysis of incidence, outcome, and associated costs of care. Crit Care Med. 2001;29(7):1303-10.

2. Dombrovskiy VY, Martin AA, Sunderram J, Paz HL. Rapid increase in hospitalization and mortality rates for severe sepsis in the United States: a trend analysis from 1993 to 2003. Crit Care Med. 2007;35(5):1244-50.

3. Shen HN, Lu CL, Yang HH. Epidemiologic trend of severe sepsis in Taiwan from 1997 through 2006. Chest. 2010;138(2):298-304.

4. Esper AM, Moss M, Lewis CA, Nisbet R, Mannino DM, Martin GS. The role of infection and comorbidity: Factors that influence disparities in sepsis. Crit Care Med. 2006;34(10):2576-82.

5. Wang HE, Shapiro NI, Angus DC, Yealy DM. National estimates of severe sepsis in United States emergency departments. Crit Care Med. 2007;35(8):1928-36.

6. Gustot T, Felleiter P, Pickkers P, Sakr Y, Rello J, Velissaris D, et al. Impact of infection on the prognosis of critically ill cirrhotic patients: results from a large worldwide study. Liver Int. 2014;34(10):1496-503.
